# Adequacy of nutritional status and dietary intake of adult and elderly patients with American Tegumentary Leishmaniasis

**DOI:** 10.31744/einstein_journal/2025AO0992

**Published:** 2025-02-14

**Authors:** Analucia Gomes Lopes Oliveira, Camila Senceite-Costa, Raquel de Vasconcellos Carvalhaes Oliveira, Marcelo Rosandisk Lyra, Benivaldo Ramos Ferreira Terceiro, Frederico Pereira Bom-Braga, Maria Inês Fernandes Pimentel, Armando de Oliveira Schubach, Patrícia Dias de Brito, Cláudia Maria Valete

**Affiliations:** 1 Laboratório de Pesquisa Clínica e Vigilância em Leishmanioses Instituto Nacional de Infectologia Evandro Chagas Fiocruz Rio de Janeiro RJ Brazil Laboratório de Pesquisa Clínica e Vigilância em Leishmanioses, Instituto Nacional de Infectologia Evandro Chagas, Fundação Oswaldo Cruz (Fiocruz), Rio de Janeiro, RJ, Brazil.; 2 Laboratório de Epidemiologia Clínica Instituto Nacional de Infectologia Evandro Chagas Fiocruz Rio de Janeiro RJ Brazil Laboratório de Epidemiologia Clínica, Instituto Nacional de Infectologia Evandro Chagas, Fundação Oswaldo Cruz (Fiocruz), Rio de Janeiro, RJ, Brazil.; 3 Instituto Nacional de Infectologia Evandro Chagas Fiocruz Rio de Janeiro RJ Brazil Serviço de Nutrição, Instituto Nacional de Infectologia Evandro Chagas, Fundação Oswaldo Cruz (Fiocruz), Rio de Janeiro, RJ, Brazil.

**Keywords:** Leishmaniasis, cutaneous, Malnutrition, Communicable diseases, Wound healing, Nutritional assessment, Eating

## Abstract

**Objective:**

Patients with American Tegumentary Leishmaniasis often experience malnutrition, weight loss, and dehydration; however, their eating profile and the degree of interference of disease manifestations on food intake is not well understood. Therefore, this study aimed to evaluate the nutritional profile and food intake of patients with American Tegumentary Leishmaniasis before treatment initiation.

**Methods:**

This was a cross-sectional study of adult and older patients with cutaneous and mucosal leishmaniasis treated at a reference center between 2011 and 2017. A nutritional consultation was conducted to collect anthropometric, biochemical, and dietary intake data (24-h dietary recalls), which were assessed for adequacy.

**Results:**

Sixty-two patients were included, with a median age of 47.5 (35.0-60.5) years, 82.3% being men, and 59.7% presenting the cutaneous form of the disease. Approximately half of the participants (51.6%) were overweight; however, most had an inadequate intake of selenium (93.5%), calcium (91.9%), magnesium (83.9%), and fiber (66.1%). Patients with mucosal leishmaniasis showed greater nutritional impairment (lower body weight, arm muscle circumference, and serum albumin levels), which was associated with older age and symptoms such as odynophagia, dysphagia, and nasal obstruction, and insufficient zinc intake than those with cutaneous leishmaniasis (p<0.05).

**Conclusion:**

Patients with mucosal leishmaniasis had greater nutritional impairment leading to reduced dietary intake and inadequate intake of micronutrients, which can negatively interfere with disease prognosis and prolong the healing process. Consequently, nutrient supplementation, a nutritional intervention, could increase the effectiveness of American Tegumentary Leishmaniasis treatment, by ensuring a good nutritional status, which is essential for tissue recovery.

## In Brief

This cross-sectional study describes the adequacy of nutritional status and food intake in patients with American Tegumentary Leishmaniasis and its relationship with the form of the disease: cutaneous or mucosal. Patients with mucosal have greater nutritional impairment associated with older age and symptoms such as odynophagia, dysphagia, and oropharyngeal lesions, which lead to reduced dietary intake and inadequate intake of micronutrients.

## Highlights

■ Most patients had an inadequate intake of selenium, calcium, magnesium, and fiber.

■ Patients with mucosal had lower body weight, arm muscle circumference, and serum albumin and zinc intake.

■ Mucosal is associated with insufficient zinc intake, low body weight, and the depletion of serum albumin and transferrin.

■ Oropharyngeal lesions are associated with calorie malnutrition and serum protein depletion.

## Graphical abstract

**Figure:**
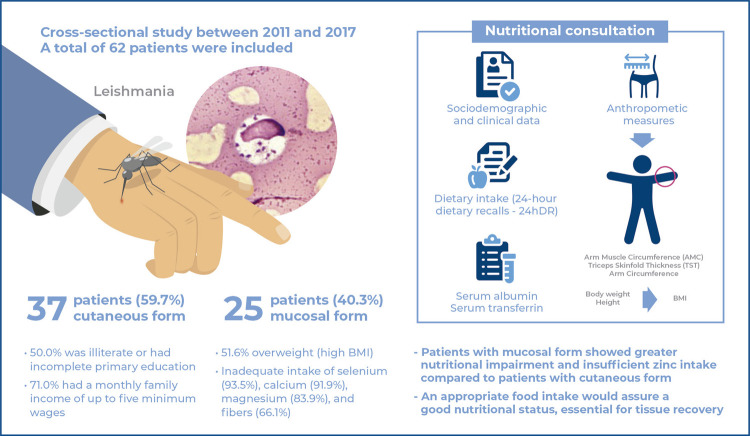

## INTRODUCTION

American Tegumentary Leishmaniasis (ATL) is an infectious parasitic disease caused by several species of protozoa of the genus *Leishmania* and is transmitted by the bite of female phlebotomine-infected sandflies. American Tegumentary Leishmaniasis has now becomean endemic in Brazil owing to geographic expansion.^1^

The disease can affect the skin, known as cutaneous leishmaniasis (CL), and mucous membranes of the upper airways and upper digestive tube, known as mucosal leishmaniasis (ML). Cutaneous leishmaniasis is the most common form, producing painless ulcers on exposed areas of the skin.^2,3^ The cutaneous lesions can remain active for several years and coexist with mucosal lesions that arise later, which is characterized as cutaneous ML (CML).^3,4^ Patients with oropharyngeal ulcers in ML often have dysphagia and odynophagia^5^ with reduced food intake and consequent malnutrition, weight loss, and dehydration. In addition to altering the smell and taste of food, nasal obstruction due to ML leads to oral breathing and altered chewing and swallowing patterns.^6^

Malnutrition, with an inadequate intake of macronutrients (proteins, carbohydrates, and lipids) and/or micronutrients (vitamins and minerals), may occur concomitantly with underweight or obesity and socioeconomic problems in developing countries.^7^ Despite the reduction in the prevalence of underweight and increase in the prevalence of overweight and noncommunicable chronic diseases, which are characteristics of the nutritional transition process, protein-calorie malnutrition remains an important public health concern in Brazil.^8^ Moreover, the relationship between protein and calorie malnutrition and immunodeficiency is well established.^9-11^ This vicious cycle is a risk factor for the development of infectious diseases, including ATL, as it influences its prognosis by limiting an individual’s ability to eat, further deteriorating their nutritional status.^9^ Nutrients are indispensable for fibroblast proliferation, collagen synthesis, and tissue repair.^10-13^

A few clinical studies have associated ATL with nutritional status. A Brazilian study^14^ showed that malnutrition (BMI <18 kg/m^2^for men and <17 kg/m^2^for women) increased the risk of developing mucosal lesions in patients with ATL. Additionally, a recent study^15^ showed that low body weight and hypoalbuminemia were associated with ML, while symptoms of impaired food intake were associated with a depleted nutritional status. Moreover, serum albumin depletion negatively affects wound healing.

However, the eating profile of patients with ATL^16,17^ and the degree of interference of disease manifestations on food intake are not well understood. Therefore, in this study, we attempt to describe the adequacy of nutritional status using various anthropometric indicators, food intake of adult and elderly patients with ATL before starting treatment, and its relationship with the form of the disease (cutaneous and mucosal) and clinical variables.

## OBJECTIVE

In this study, we aimed to explore the association among nutritional status, food intake, and clinical form, and identify patients with greater nutritional impairment.

## METHODS

This was a cross-sectional analysis of a longitudinal study involving 62 adult and older patients before starting treatment at the *Instituto Nacional de Infectologia Evandro Chagas* (INI/Fiocruz), a reference unit for the treatment of infectious diseases in Rio de Janeiro, between 2011 and 2017. The diagnosis of ATL was confirmed using one or more of the following methods: isolation of promastigotes in culture, visualization of amastigotes on direct examination or histopathological examination, or detection of parasitic DNA by PCR. The study was approved by the Ethics Committee of INI/Fiocruz (IRB 0016.0.009.000-02). All patients signed an informed consent form and were included and evaluated prior to starting treatment.

Sociodemographic, clinical, anthropometric, and dietary data were collected during the nutritional consultation. The professional activities of the patients were classified as extra-domestic (active workers and students) or domestic (domestic, retired, or unemployed) for the analysis. Variables such as the clinical form of leishmaniasis, location of lesions, and presence of oral ulcers were collected from medical records.

Body weight and height were measured on an electronic platform digital scale (Filizola^®^) to calculate Body Mass Index (BMI). Patients were classified in presence or absence of underweight, according to BMI and age: BMI <18.5 kg/m^2^ for adults (from 20 - 59 years old) and BMI <22 kg/m^2^ for older individuals (≥60 years old).^18^ The Triceps Skinfold Thickness (TST) and Arm Circumference (AC) were measured using an inelastic measuring tape and a digital plicometer, respectively, to calculate Arm Muscle Circumference (AMC). Patients were classified as having protein or caloric malnutrition by comparing the AMC and TST measurements, respectively, with a normality pattern according to sex and age.^19,20^ Depletion of serum proteins (albumin and transferrin) was considered according to the following cutoff points: ≤3.5 g/dl for albumin and ≤200 mg% for transferrin.^21^

Dietary intake was assessed using 24-h dietary recalls (24hDR), in which a trained nutritionist requested the patients to report all foods and beverages consumed the previous day using a photo album, following a structured and systematized interview (Automated Multiple-Pass method).^22^ One to two face-to-face 24hDRs were performed for each patient, with an interval of up to 20 days. Portions of each food reported in household measurements were converted to milligrams/grams and/or milliliters/liters, and the data were entered into a nutritional analysis software (Nutriquanti program, São Paulo, SP, Brazil).^23^ Adequacy of dietary intake was determined by comparing the average intake of each nutrient with the estimated average requirement (EAR), recommended for estimating the prevalence of inadequacy in groups. When EAR was not available, the use of adequate intake (AI) was recommended according to the Dietary Reference Intake (DRI).^24^ Macronutrients (proteins, carbohydrates, lipids, and fatty acids) were adjusted using the energy density method and expressed as a proportion of energy intake (E%). Fiber intake was expressed in g/1000 kcal using the following formula: total fiber (g) × 1000/total energy.

The frequencies of the categorical variables and summary measures (median and interquartile range [IQR]) of the quantitative variables were estimated. The normality of the quantitative variables was rejected using the Shapiro-Wilk normality test. Differences in the distribution of quantitative variables according to the clinical form of leishmaniasis were evaluated using the non-parametric Mann-Whitney *U* test. The association between categorical variables was investigated using Pearson’s χ^2^ test or Fisher’s exact test (when the number of observations in a group was <5). Statistical significance was set at p<0.05. Statistical Package for Social Sciences (SPSS) version 16.0 (IBM Corp, Armonk, NY) was used for data analysis.

## RESULTS

Sixty-two patients with a median age of 47.5 (35.0-60.5) years were included, most of whom were men (n=51, 82.3%). Half of the cohort (50.0%) was illiterate or had incomplete primary education, 71.0% had a monthly family income of up to five minimum wages (each minimum wage corresponding to US$ 296.15, Central Bank of Brazil, 2018), and 73% had extra-domestic professional activity.

Thirty-seven patients (59.7%) had CL and 46.7% had a lower limb injury. Twenty-five patients (40.3%) had ML; 92.0% in the nasal mucosa, and 44.0% in the oral mucosa. The signs and symptoms reported by the patients were nasal obstruction (37.1%), recent reduction in food intake (31.1%), dyspnea (25.8%), dysphagia (8.1%), constipation (8.1%), odynophagia (6.5%), anorexia (6.5%), nausea (6.5%), abdominal pain (4.8%), and vomiting (1.6%). Only patients with ML reported nasal obstruction, dysphagia, or odynophagia.

**Figure 1:**
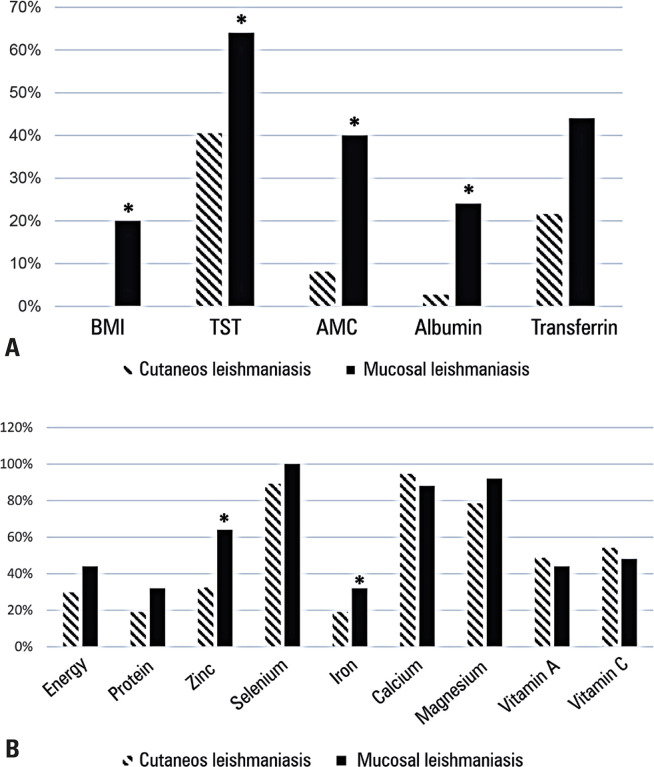
Comparison of the frequency of nutritional inadequacies - anthropometric (A) and nutrient intake (B) - between patients with cutaneous and mucosal leishmaniasis

The median BMI of the patients was 25.5 (21.7-28.7) Kg/m^2^, with 32 patients (51.6%) classified as overweight and/or obese, and 8.1% (n=5) as underweight. However, 22.6% and 51.6% patients had protein and caloric malnutrition, respectively. Median serum albumin was 3.9 (3.7-4.2) g/dl and 12.1% of patients had hypoalbuminemia. Transferrin depletion was detected in 47.5% of the patients, with a median of 200.6 (177.4-222.8) mg%. Patients with ML had a lower body weight, AMC, and serum albumin levels (Table 1), whereas the frequency of nutritional status inadequacies was higher than those with CL (Figure 1A).

**Table 1:** Comparison of the medians of the nutritional assessment variables between patients with cutaneous leishmaniasis or mucosal leishmaniasis

	Cutaneous leishmaniasis (n=37) median (IQR)	Mucosal leishmaniasis (n=25) median (IQR)
Body weight (kg)	79.1 (69.1-90.1)	63.40 (55.6-75.1)*
BMI (kg/m^2^)	27.1 (23.9-29.8)	22.9 (19.8-28.5)
TST (mm)	12.3 (9.6-16.9)	8.4 (5.6-15.2)
AMC (cm)	28.6 (26.3-30.9)	25.0 (22.6-27.4)*
Albumin (g/dl)	4.0 (3.8-4.2)	3.7 (3.5-3.9)*
Transferrin (mg%)	205.8 (178.6-225.0)	187.4 (169.4-201.4)

*p<0.05 by Mann-Whitney U test.

IQR: interquartile range; BMI: body mass index; TST: triceps skinfold thickness; AMC: arm muscle circumference.

* p<0.05 by Mann-Whitney test.

Patients ingested a median of 2163.1 (1431.3-2773.1) total kcal or 30.8 (21.5 - 38.9) kcal/kg. In terms of the energy contribution of macronutrients, the median protein, carbohydrate, lipid and fiber intake was 16.0% (12.9-19.1), 54.0% (45.7-58.2), 32.4% (29.1-37.6) and 11.9 (8.9-16.7) g/1000 kcal, respectively. However, 41 patients (66.1%) had insufficient intake. With regard to micronutrients, most patients had poor intakes of selenium (93.5%), calcium (91.9%), magnesium (83.9%), and fiber (66.1%) (Table 2).

**Table 2:** Median micronutrient intake of 62 patients with American tegumentary leishmaniasis, according to the requirements of the Dietary Reference Intakes

Micronutrients	Requirements DRI	Median (IQR)	Insufficient intake n (%)
Vitamin A (mcg)			
Men	625	680.2 (193.3-1382.2)	29 (46.8)
Women	500		
Vitamin C (mg)			
Men	75	74.2 (19.9-147.0)	32 (51.6)
Women	60		
Zinc (mg)			
Men	9.4	10.2 (5.4-16.1)	28 (45.2)
Women	6.8		
Selenium (mcg)	45	6.7 (2.5-16.4)	58 (93.5)
Iron (mg)			
Men	6	10.1 (6.2-14.3)	15 (24.2)
Women	5-8.1		
Calcium (mg)	1000-1200	427.7 (282.1-618.6)	57 (91.9)
Magnesium (mg)			
Men	330-350	234.5 (151.2-294.9)	52 (83.9)
Women	255-265		

ND: not determined; IQR: interquartile range.

All patients with ML had a higher frequency of inadequate selenium (n=25) and zinc intake (64.0% *versus* 32.4% CL, p=0.014) (Figure 1B). Additionally, the mean iron intake was lower in patients with ML than in those with CL (Table 3). Inadequate zinc intake was also associated with nasal obstruction (62.5% *versus* 26.3% without obstruction; p=0.002) and dyspnea (68.8% *versus* 28.9% without dyspnea; p=0.005).

**Table 3:** Comparison of the median intake of macro and micronutrients between cutaneous leishmaniasis and mucosal leishmaniasis

	Cutaneous leishmaniasis (n=37) Median (IQR)	Mucosal leishmaniasis (n=25) Median (IQR)
Proteins (%E)	15.8 (12.5 - 19.3)	16.1 (14.2 - 19.1)
Carbohydrates (%E)	49.8 (40.1 - 58.0)	56.2 (49.2 - 59.4)
Lipids (%E)	33.5 (30.1 - 39.4)	30.9 (27.7 - 34.2)
Energy (kcal/kg)	30.9 (22.7 - 36.7)	26.9 (18.9 - 40. 9)
Fibers (g/1000kcal)	13.5 (8.1 - 18.5)	10.0 (8.9 - 14.4)
Vitamin A (mcg)	669.2 (255.2 - 2114.6)	707.7 (177.1 - 1060.2)
Vitamin C (mg)	73.9 (16.9 - 133.8)	77.6 (38.2 - 163.6)
Zinc (mg)	13.4 (7.7 - 18.2)	8.0 (4.6 - 11.2) *
Selenium (mcg)	7.1 (3.5 - 20.9)	6.1 (1.6 - 13.4)
Iron (mg)	11.9 (7.4 - 15.9)	9.1 (5.1 - 12.5) *
Calcium (mg)	440.2 (284.5 - 587.3)	415.1 (252.5 - 742.0)
Magnesium (mg)	230.3 (153.2 - 307.4)	241.3 (134.4 - 271.5)

IQR: interquartile range; %E: proportion of energy consumption; kcal/kg: kilocalories per kilogram of weight; g/1000 kcal: grams per 1000 kilocalories; mcg: micrograms; mg: milligrams.

*p<0.05 by Mann-Whitney U test.

A significant association was observed between ML and low body weight (100% ML *versus* 35.1%, p=0.008), and age (80.0% elderly *versus* 21.1% adults, p=0.014). Additionally, caloric malnutrition was associated with the presence of oral and pharyngeal lesions (25.2% *versus* 0% without lesions; p=0.003) and recent reduction in dietary intake (45.2% *versus* 16.7% without reduction; p=0.016). Moreover, patients with ML had a higher frequency of protein depletion (71.4% *versus* 31.2% CL; p=0.009), nasal obstruction (64.3% *versus* 29.4% without obstruction; p=0.017), dyspnea (50.0% *versus* 14.8% without dyspnea; p=0.021), and recent reduction in food intake (57.1% *versus* 23.4% without reduction; p=0.022) compared to those with CL.

In the biochemical evaluation, we observed associations between ML and hypoalbuminemia (85.7% *versus* 35.7%; p=0.015), dyspnea (85.7% vs. 20.0%; p<0.001), oral (43.6% *versus*. 8.3%; p=0.032), pharyngeal lesions (43.2% *versus* 10.6%; p=0.048), and a recent reduction in dietary intake (71.4% *versus* 28.0%; p=0.035). A significant association between ML and serum transferrin depletion (57.9% *versus* 28.6%; p=0.046), oral lesions (26.3% *versus* 0%; p=0.012), and pharyngeal lesions (32.5% *versus* 5.2%; p=0.026) was also observed.

## DISCUSSION

In this study, the assessment of the nutritional status and dietary intake of 62 patients with ATL showed that although approximately half of the cohort was overweight, depletion of nutritional components occurred in most patients. Most of them had an inadequate intake of fiber, magnesium, calcium, and selenium. Furthermore, patients with ML had a more compromised nutritional status and insufficient intake of selenium and zinc than those with CL. These demographic characteristics of the cohort, mostly male adults, are consistent with those reported in previous studies^5,15,25-28^ and can be explained by the professional activities of men, characterizing ATL as an occupational disease.^15,29,30^

The cohort had a high prevalence of patients with incomplete education up to elementary school and a monthly family income of up to five times the minimum wage. These results are consistent with the distribution of leishmaniasis in the poorest segments of the global population.^1,2^

Studies have shown that symptoms, such as nasal obstruction, dyspnea, dysphagia, and odynophagia, result from mucosal lesions in the nasal, oral, pharyngeal, and laryngeal cavities.^5,27^ The greater frequency of reduced food intake observed in patients with ML has been described as a consequence of the presence of oral lesions and the associated symptoms of dysphagia and odynophagia, leading to malnutrition.^15^

Despite the low prevalence of underweight, we observed high frequencies of inadequacy in somatic and visceral proteins (hypoalbuminemia and hypotransferrinemia) and adipose compartments. In a study conducted in Bolivia, no difference was observed in the nutritional parameters between patients with CL and healthy individuals, suggesting that the cutaneous form is not directly associated with impaired nutritional status.^17^ Therefore, we believe that the discrepancy regarding the prevalence of somatic protein and adipose compartment depletion observed previously compared with the current study was the small sample size. Additionally, the present study highlighted that ML is associated with a more inadequate nutritional status (lower body weight and impairment of the somatic and visceral protein compartments), possibly due to injuries and symptoms, such as dysphagia, nasal obstruction, and dyspnea, confirming previous results.^5,15^

Regarding the energy contribution of macronutrients, we observed a value lower than the national average for the percentage of proteins (16.01% *versus* 19%) and carbohydrates (54.02% *versus* 57%) and a higher value for lipids (32.42% *versus* 30%).^31^ Moreover, the low fiber intake observed in this study is consistent with the change in the Brazilian population’s diet: lower consumption of rice, beans, and fruits, and an increase in the consumption of industrialized foods.^31^

Most patients, especially those with ML, have insufficient intake of selenium, magnesium, and calcium, nutrients that are involved in wound healing,^13,21^ the formation of antibodies, and the development of the immune system.^13,21^ However, as only 8.1% of the patients in our study were underweight, we believe that this insufficient intake of nutrients is a consequence of a low caloric intake but of the low density of nutrients in the foods consumed more frequently, characterizing hidden hunger or a lack of micronutrients.^7^ Generally, selenium intake by the population is below the requirement because, in addition to the low content and bioavailability of selenium in foods, its main sources are animal foods, which have a higher cost. Consequently, the frequency of beef consumption among the Brazilian population in both rural and urban areas has reduced.^31^ While a previous study in Bolivia reported that patients with CL had dietary inadequacies that were not associated with the disease.^17^ A more recent study carried out in Iran showed that insufficient food intake in patients with CL interfered negatively with the clinical course of the disease.^16^

Our results showed that ML is associated with zinc deficiency. Whereas Lazarte et al^17^ reported that deficient intake of this nutrient was associated with CL. Deficient zinc intake is also associated with nasal obstruction and dyspnea, which are frequent symptoms of ML. Oral breathing, resulting from nasal obstruction, further leads to a decrease in smell and taste, changes in the pattern of chewing and swallowing^6^ and may interfere with the ingestion of protein foods (sources of zinc), as they are more fibrous. As patients with ML presented greater nutritional impairment and the presence of oropharyngeal and nasal ulcers reduced food intake, these patients would require appropriate nutrient intake, especially zinc and selenium.

The frequency of underweight was higher in the older group, which is consistent with a previous study.^15^ This impairment in nutritional status may occur because of expected muscle loss in older individuals, including decreased muscle strength or sarcopenia. Another possible cause is the presence of physiological alterations such as xerostomia, heartburn, reduced tongue tone, and muscular hypotonia of the mouth and pharynx, which can alter chewing and swallowing and lead to reduced appetite. In addition to the expected physiological causes of aging, previous studies have reported an association between advanced age and ML.^5,15,28^ Therefore, considering that mucosal lesions are more frequent in this age group and cause symptoms that reduce dietary intake, leading to malnutrition, malnutrition is expected to occur more frequently in older individuals.

Notably, we observed greater nutritional impairment in patients with ML because this form has several clinical manifestations that negatively interfere with dietary intake, leading to malnutrition. In addition to the association of ML with low weight and hypoalbuminemia, as previously observed^15^ we report, for the first time, the association of this clinical form and the presence of oropharyngeal lesions with protein-somatic malnutrition and serum transferrin depletion. This nutritional compromise can lead to less favorable therapeutic results in these patients, as these serum proteins play a fundamental role in healing, promoting tissue growth, and cell renewal.^10^ The presence of an oral lesion with reduced dietary intake and consequent malnutrition has also been associated with a longer healing time in ML.^5^

Despite its positive outcomes, this study has some limitations. First, we could not specify whether nutritional impairment predated ATL because patients were included in the study at the time of diagnosis or initiation of treatment and not at the onset of symptoms. Second, the assessment of dietary intake through recall does not reflect the usual consumption of the participants; therefore, it may not reflect persistent dietary inadequacies. Third, this was a descriptive study of patients evaluated sequentially during the study period at an outpatient clinic, and the small sample size did not allow comparison of measurements in subgroups or power analysis. Finally, the lack of a Control Group did not allow for adequate comparison. Although we were unable to perform a sample calculation or regression analysis, we believe that our results can help in the design of future studies, and a model adjustment would be necessary to confirm some findings. The measurement of serum micronutrients is an objective measure for identifying nutritional deficiencies, regardless of the patients’ dietary intake.

## CONCLUSION

Patients with mucosal leishmaniasis had greater nutritional impairment associated with older age and symptoms, such as odynophagia, dysphagia, and nasal obstruction, which led to reduced dietary intake. Consequently, we observed an inadequate micronutrient intake, which could negatively interfere with wound healing. Therefore, longitudinal monitoring of the nutritional status and dietary intake of patients with American Tegumentary Leishmaniasis is necessary until a clinical cure is achieved. We suggest that nutritional interventions, such as nutrient supplementation, could increase the effectiveness of American Tegumentary Leishmaniasis treatment, as an appropriate food intake would ensure a good nutritional status, which is essential for tissue recovery.
